# Characteristics of Ancient Ship Wood from Taicang of the Yuan Dynasty

**DOI:** 10.3390/ma16010104

**Published:** 2022-12-22

**Authors:** Xinyou Liu, Xin Xu, Xinwei Tu, Wanrong Ma, Houyi Huang, Anca Maria Varodi

**Affiliations:** 1 Co-Innovation Center of Efficient Processing and Utilization of Forest Resources, Nanjing Forestry University, Nanjing 210037, China; 2 College of Furnishing and Industrial Design, Nanjing Forestry University, Str. Longpan No.159, Nanjing 210037, China; 3 Faculty of Furniture Design and Wood Engineering, Transilvania University of Brașov, 500036 Brasov, Romania; 4 Advanced Analysis and Testing Center, Nanjing Forestry University, Str. Longpan No.159, Nanjing 210037, China

**Keywords:** microscopy, waterlogged archaeological wood, SEM, XRD, FTIR, nanoindentation

## Abstract

In this study, wood samples extracted from the Taicang ancient ship, dating back to the Yuan Dynasty, were investigated to study the characteristics of waterlogged archaeological wood. The macroscopic characteristics and microscopic structures were used to identify the wood species. To assess the degree of degradation of the waterlogged archaeological wood, X-ray diffraction (XRD), nanoindentation (NI), Fourier transform infrared spectroscopy (FTIR), and scanning electron microscopy (SEM) were used to compare the new and ancient wood samples from the same species. The microscopic structures of the samples were identified as *Pinus massoniana*. The XRD and nanoindentation results revealed that the crystallinity index of the cellulose decreased from 41.07% to 33.85%, the elastic modulus was reduced by 20.90%, and hardness was reduced by 55.6% compared with the new wood. The FTIR spectra revealed that biological deterioration occurred in the cellulose and hemicellulose, but there was no noticeable change in the lignin content. These results provide helpful information for the conservation and restoration of ancient ships.

## 1. Introduction

Ancient boats and ships were an essential mode of transportation in ancient waters and a means of exploring ancient sea civilizations [1,2]. Ancient China was a vast seafaring nation. China established the “Maritime Silk Road” to conduct maritime trade with neighboring countries as early as the Qin and Han Dynasties [3,4,5]. To date, more than 50 ancient ships have been unearthed in China [6,7,8]. These ancient ships were mostly made of wood, including coniferous wood types such as Cunninghamia sp., Cupressus sp., Picea sp., and Pinus sp., and also some hard wood types (Populus sp., Quercus sp., Styphnolobium sp., Tectona sp., and Ulmus sp.) [9]. If these kinds of wood are in a state of high humidity or water saturation for a long time, they can be easily attacked by fungi or bacteria [10], resulting in a fluffy structure and huge moisture content [11,12]. The cellulose, hemicellulose, and lignin in the wood are degraded and lost, leading to loose and reduced strength for the wood, which will be prone to deformation and other defects after excavation exposure to air [13,14,15,16]. The conservation of excavated ancient ships and restoration of their original appearance are critical issues confronting cultural relic conservationists. However, studying the characteristics of ancient waterlogged trees is the basis for the restoration and conservation of ancient ships.

Ancient ship wood research has recently produced valuable findings [17,18,19,20]. However, before preserving the archaeological wood, its deterioration must be evaluated. This can be achieved by studying the wood density, cellulose microfibril angle [21,22,23], water absorption, molar mass of the holocellulose, rate of decay [24,25,26], and ultrastructure of the cell wall [27]. In addition, the accumulation of sulfur in underwater or underground environments accelerates the deterioration of wood, and desalination and desulfurization are essential steps in restoring and conserving ancient ship wood [28,29]. Given the fragility of the cell walls of degraded ancient ship wood, ethanol replacement, PEG replacement, freeze–vacuum drying, supercritical carbon dioxide, and other unique drying methods must be used [30,31,32,33]. PEG, trehalose, polyoctadecanol, epoxy resin, and other materials with good stability have been widely used in ancient wood consolidation [34,35,36,37,38,39]. In addition, scanning electron microscopy (SEM) [40,41], X-ray diffraction (XRD) [42], infrared spectroscopy (FTIR) [43], X-ray photoelectron spectroscopy (XPS) [44], thermogravimetric analysis (TGA) [45], and nanoindentation (NI) [46,47,48] techniques are essential methods for effectively evaluating the restoration and protection effects of ancient ship wood. In short, the research on the characteristics of ancient ship wood is the foundation and premise of the above research findings, which are critical for the restoration and preservation of ancient ships.

**Figure 1:**
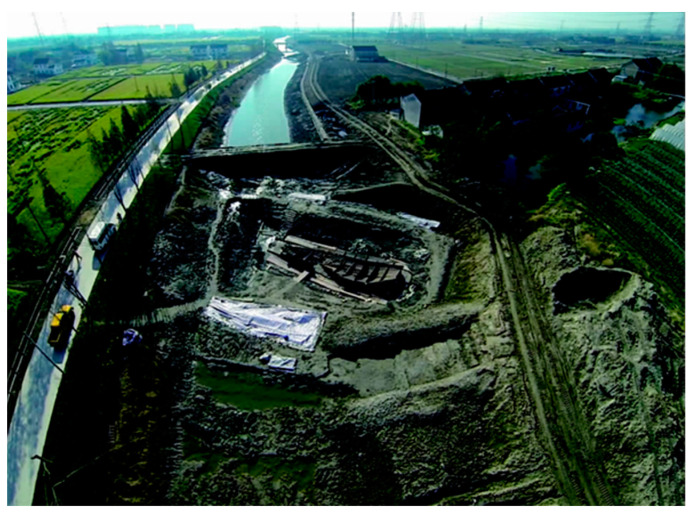
The ancient ship excavation site.

During a draining project in May 2014, a sunken wooden ship was discovered in the Wanfeng Village section of the Banjing River in Taicang City (see Figure 1). From August to December 2014, the Nanjing and Taicang Museums conducted an archaeological excavation and rescue of the sunken ship and relocated it to the Haifeng farm in Wanfeng Village for cultural relic protection. Several clay porcelain specimens were discovered during the archaeological excavation of ancient wooden ships. Based on the porcelain fragments unearthed in the silt of the river, it was preliminarily inferred that the ancient boat belonged to the Yuan Dynasty. The Banjing River is a vital branch of Liujia Port in Taicang. The Liujia port, the famous “six states wharf” of the Yuan Dynasty, is the starting point of the grain transport from the south to the Yanhai Sea [49,50].

This study aimed to analyze the characteristics of the ancient ship. The microscopic structure was investigated to detect wood species. A variety of analyses, including Fourier transform infrared spectroscopy (FT-IR), X-ray diffraction (XRD), and scanning electron microscopy (SEM), were employed to identify the differences between new and ancient woods. The present study offers significant instructions for creating artificial ancient woods to restore and replace destroyed wood in ancient wooden ships in China.

## 2. Materials and Methods

### 2.1. Materials

In this study, wood samples were taken from a part of the keel in the no. 8 hatch of the ancient Taicang ship, obtained from the Taicang Museum. The size of this wood block was approximately 50 mm × 65 mm × 350 mm, and the growth ring was visible in the cross-section. The surface of the wood appeared grayish-black owing to the deterioration. Moreover, the initial moisture content reached 65.3 ± 1.7% (measured according to standard GB/T 1931 (2009)) [51]. Finally, the block was dried in an electric oven at 40 °C until the moisture content decreased to 10%.

### 2.2. Microscopic Identification

The macroscopic characteristics of the samples were determined using a 10× magnifying glass to identify the wooden species more accurately. As a result, it was possible to conclude that the samples had well-defined annual rings and a narrow band of latewood. In addition, the wood had a grey, deteriorated surface that had been cleaned, revealing a yellow-reddish hue. Therefore, it was identified as a softwood.

The extracted wood was boiled in distilled water for approximately 6 h, or until the sample sank to the bottom of the water, to soften the sample. Then, using a microtome, the sample was cut into 30 µm slices in the transverse, radial, and tangential directions. Before observation, the sections were stained with saffron and rinsed with water to increase the contrast of the microscopic images. Thin, stained, transparent slices were observed in transmitted light at varying magnifications (40 to 100×) under an optical microscope (Zeiss Axio Scope A1 microscope, Carl Zeiss AG, Oberkochen, Germany) with AxioVision Rel.4.8 (Carl Zeiss AG, Oberkochen, Germany).

### 2.3. X-ray Diffraction

Based on the aforementioned microscopic identification results, new wood samples of the same species were prepared, and the changes in cellulose crystallinity in the ancient wood samples were compared. After drying, the new and ancient wood samples were ground to an 80-mesh powder and pressed at room temperature into ten sample sheets (five new and five ancient wood samples). In situ XRD was performed on the wood samples using an X’Pert Pro multipurpose diffractometer (PANalytical, Almelo, Netherlands) and a Rigaku Smart Lab 9 kWXRD system (Shimadzu Corporation, Kyoto, Japan). Furthermore, θ–2θ scanning was used to assess the scattering intensity and angle. The scanning speed was 2°/min and the angle range was 5–60°. The provided spectrum was the average of five measurements for new and ancient woods. The height of the (002) peak (I_002_, 2θ = 22.8°) and the minimum value between the (002) peak and (101) peak (IAM, 2θ = 18°) were used to determine the crystallinity of the cellulose using the Segal method [52], and the equation was as follows:

CR_x_ = (I_002_ − I_AM_)/I_002_ × 100% (1)

where CRx (%) represents the crystallinity index of the cellulose, I_002_ is the diffraction peak intensity corresponding to the (002) lattice plane at 2θ = 22.8° and Iam is the diffraction intensity at 2θ = 18°.

### 2.4. Chemical Structure Analysis

Changes in the chemical structures of both new and ancient wood samples were investigated. The FTIR test equipment was an IR spectrometer (Tensor 27, Bruker, Ettlingen, Germany) with a spectral resolution of 4 cm^−1^ between 4000 cm^−1^ and 400 cm^−1^ for a total of 32 scans. Before the measurement, the background spectra were collected after the light equipment was aligned. The spectra were described as a means of sextuple measurements for each wood sample.

### 2.5. Quasi-Static Nanoindentation Test

The dimensions of the wood specimens (ancient and new) to be nanoindented were 7 mm × 5 mm × 5 mm. The bottom and top of the prepared blocks were perpendicular to the longitudinal axis of the cell wall and parallel to each other. All samples were placed on the platform, and all angles were verified with a straight edge at 90°. Next, the samples were attached to a metal sample holder as pyramids. Finally, the apex was smoothed with a diamond knife using an ultramicrotome. The specimens were prepared using the procedure described by Meng et al. [53]. The nanoindenter was an in situ nanomechanical test system (Hysitron TI980, Bruker, Ettlingen, Germany) with a diamond Berkovich tip operated in open-loop control. The experiment was carried out in load function mode using a three-segment load ramp; the load application lasted for 5 s, the hold time was 5 s, and the unloading time was 5 s. The peak load was 400 N for all the indents in the test, leading to a maximum penetration depth range of 250–300 nm. Five indentations for both ancient and new wood samples were carried out based on the ambient temperature and relative humidity. Based on the nanoindentation theory, the hardness (H) and reduced elastic modulus (Er) were computed from the load–displacement data using the equations below, as depicted by Oliver and Pharr [54].

The hardness of the sample was computed as follows:

(2) $$H=\frac{P_{max}}{A}$$

where *Pmax* is the peak load determined at a maximum depth in the indentation cycle, and A is the projected contact area between the indenter and sample. The *t*-test in SPSS (Statistical Product Service Solutions, IBM Corp., IBM SPSS Statistics for Windows, v. 25, Armonk, NY, USA), was used to compare the treatments (*p* = 0.05).

The reduced elastic modulus (*Er*) of the sample was computed as follows:

(3) $$E_{r}=\frac{\sqrt{\pi }}{2}\frac{S}{\sqrt{A}}\;\;$$

where *A* denotes the projected contact area and *S* (stiffness) denotes the slope of the unloading curve in the load–displacement graph. For the computation of *S*, a linear approximation was employed for the high-load part of the unloading curve (90–70% alterations in load). The *t*-test in SPSS was used to compare the treatments (*p* = 0.05).

### 2.6. Morphological Characteristics

The surface morphologies of the wood specimens were monitored in this study using environmental SEM (Quanta 200, FEI, Eindhoven, Netherlands) through electrical conductivity determination tests to examine possible physical property variations in the ancient and new wood samples to explore their physical architectures. A 2 nm sputter gold coating was applied to the wood specimens using gold–palladium SEM annular sputtering, with the target being SC502–314 with dimensions of 2″ ID × 3″ OD × 0.1 mm (Quorum Technologies, Watford, UK). The bombarding voltage was set to 20.0 kV during the SEM.

## 3. Results

### 3.1. Microscopic Identification

The macroscopic aspects of wood serve as a foundation for its identification. The wood surface had a greyish-black color owing to the deterioration. After the deteriorated layer was removed, the earlywood (yellow) and latewood (reddish) colors were noticeably different, with a distinct turpentine odor. The annual ring delineation was clear. The early wood belt, which comprises approximately 2/3–3/4 of all annual rings, was relatively broad. Tracheids were visible under a magnifying glass, and the transition between early and late wood types was abrupt. No axial parenchymal wood rays were visible in the transverse section with a magnifying glass, but they were visible in the radial section with the naked eye.

**Figure 2:**
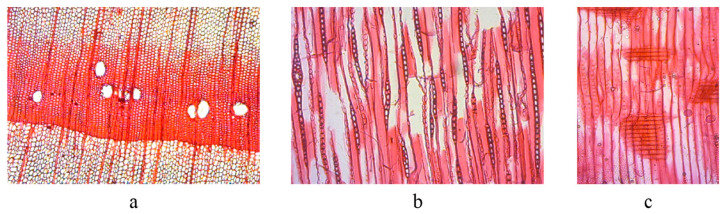
Micrographs of the investigated sample in the transverse (**a**), tangential (**b**), and radial (**c**) sections.

The tracheids of earlywood were rectangular and polygonal under the microscope in the transverse section, the axial resinous channels were large (Figure 2a), and the wood rays were thin. There were two types of wood rays: those with single-ray heights of 1–20 cells or more, and those containing 5–15 cells. Spindle rays have radial resinous channels, three or two rows of ray cells below the proximal canal, gradually taper to a single row at the upper and lower ends, and are1–10 cells high or more. The ray cells were elliptical, long elliptical, rectangular, and oval, with a small amount of resin (Figure 2b). The pit was pane-shaped and sparse in the cross-field between the ray parenchyma cells and earlywood tracheids. The lipid-secreting cell walls of the resinous channels were thin and frequently contained impenetrable bodies. The inner wall was deeply serrated and had a wavy outer edge (Figure 2c). Based on the above structural characteristics, through the comparison of the relevant data [50,55], wood specimens, and wood microsections, the wood was identified as a hard pine wood of either *Diploxylon*, *Pinaceae*, or *Pinus*. However, according to the distribution of tree species and wood habits, *Pinus massoniana* was more likely, which has been also found from some ancient shipwrecks in China [9,10].

### 3.2. X-ray Diffraction

**Figure 3:**
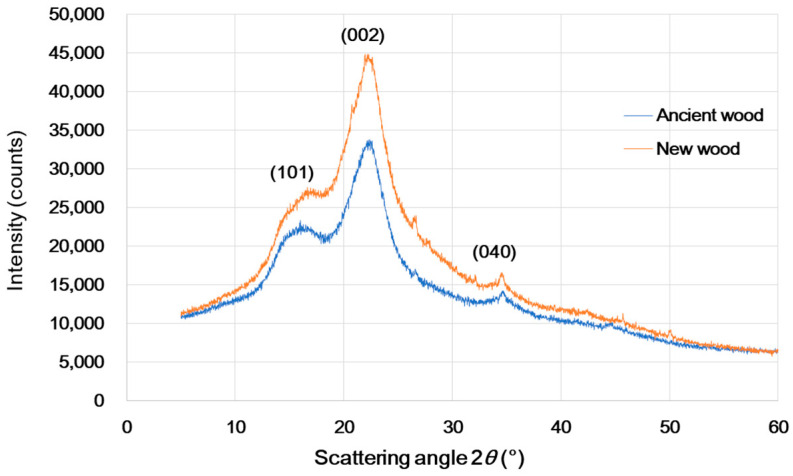
X-ray diffraction patterns of the ancient and new wood samples.

One of the three main constituents of wood, cellulose, which serves as the skeletal material for wood cells, is divided into crystalline and amorphous zones. The cellular crystallinity is known as the proportion of crystalline to amorphous and crystalline areas in cellulose microfilaments. The crystallinity of wood cellulose is frequently determined using an X-ray diffractometer. Three diffraction peaks corresponding to the (101), (002), and (040) crystal facets appeared at 2θ of 18°, 22.5°, and 35°, respectively, as shown in Figure 3. A diffraction peak of (002) in the 2θ = 22.8° vicinity exhibited the maximum value, whereas an exceedingly small value was noted in the vicinity of 2θ = 18°. Drastically weakened diffraction peak intensities were observed for the ancient *Pinus massoniana* wood. This was because massive amounts of cellulose were lost. The crystallinity index of the cellulose was estimated using Equation (1) to be 41.07% for new wood and 33.85% for ancient wood. The decline in crystallinity of the ancient wood was attributable to the cellulose loss [56].

### 3.3. Chemical Structure Analysis Using FTIR Spectroscopy

**Figure 4:**
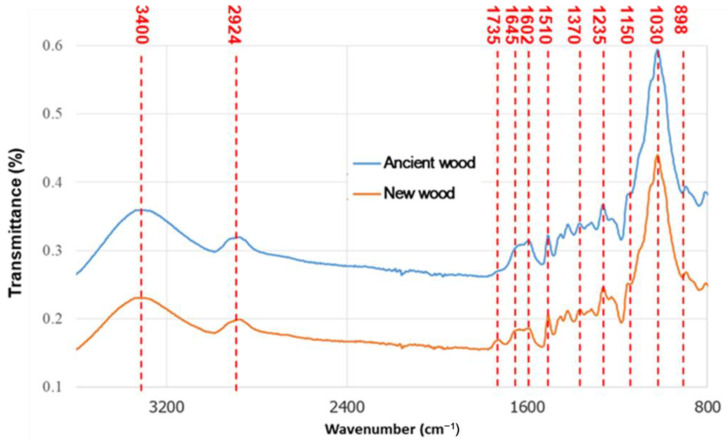
FTIR spectra of the new and ancient wood samples.

The degradation of the ancient ship wood in underground environments caused its chemical composition to change. FTIR spectroscopy is commonly used to detect chemical changes in wood. Figure 4 compares the infrared spectra of the ancient and new wood samples. The spectral band of the ancient wood was slightly reduced at 3400 cm^−1^, which corresponded to the stretching vibration of hydroxyl (-OH) [57,58,59], indicating that dehydration may have occurred during the deterioration of the ancient wood. Furthermore, the FTIR peak at 1735 cm^−1^ was attributed to the C=O stretching vibration of xylan. In contrast to the new wood spectrum, the ancient wood spectrum lacked an absorption peak at 1735 cm^−1^, which was attributed to the stretching vibration of the xylan acetyl group C=O double bond (CH_3_C=O) [60,61]. This implied low concentrations of cellulose and hemicellulose in the ancient wood specimens. In contrast, the peaks at 1602 cm^−1^ conforming to C=C stretching of the aromatic ring (lignin) increased slightly [62,63], while the peaks at 1510 cm^−1^ did not change significantly, indicating that the content of lignin in the ancient wood increased [11,43]. This implied that lignin was only slightly degraded during the deterioration process, which is consistent with the previous literature [64].

### 3.4. Nanoindentation Test

**Figure 5:**
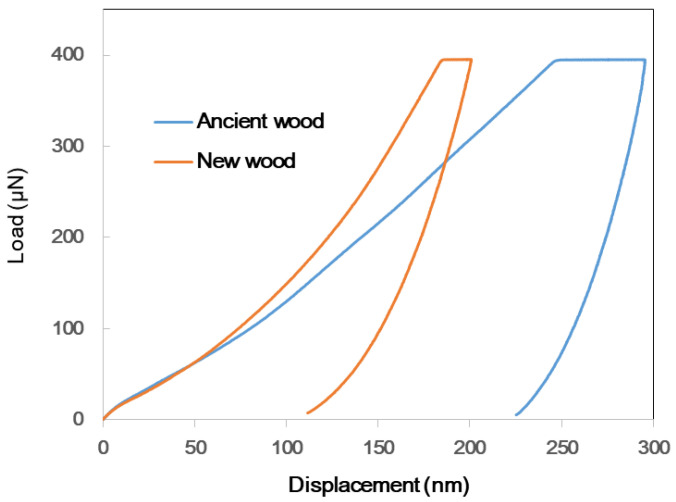
Typical NI load–displacement curves of ancient and new wood samples.

The nanoindentation test successfully measured the mechanical changes in wood load–displacement data at the microscale and the measured wood’s hardness and elastic modulus at the submicron scale [52]. Figure 5 illustrates the typical NI load–displacement curves of the ancient and new wood samples. Table 1 presents the elastic modulus and hardness of the ancient and new wood samples. The elastic modulus represents the ability of the material to resist elastic deformation. The Er value and hardness value of the new wood were 7.57 GPa and 0.45 GPa, respectively, while the values of the ancient wood were 5.99 GPa and 0.20 GPa. The results indicated that the degradation of cellulose and hemicellulose in the wood cell walls resulted in significant decreases in the mechanical strength and hardness by 20.9% (*p* < 0.0001) and 55.6% (*p* < 0.0001), respectively.

### 3.5. Morphology

**Figure 6:**
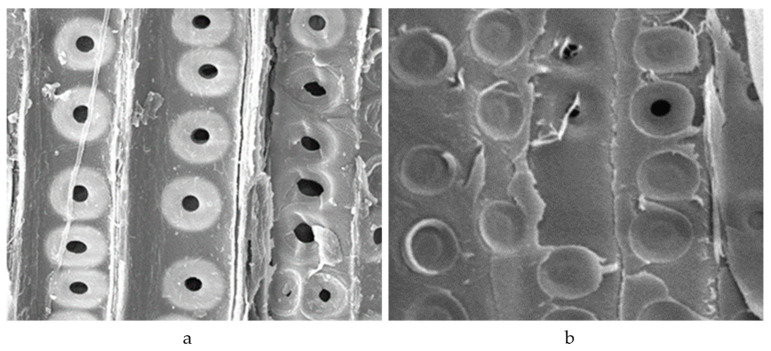
SEM images of ancient and new wood samples at 1000× magnification: (**a**) ancient wood; (**b**) new wood.

Figure 6 shows SEM images comparing new and ancient wood samples to assess potential changes in the physical structure of the ancient wood after deterioration. When compared to the microscopic image of the new wood on the right in Figure 6, the majority of the grain holes in the ancient wood on the left were broken, and there were cracks on the tracheid wall, indicating that the degradation of cellulose and hemicellulose in the wood resulted in a change in its physical structure.

## 4. Conclusions

In conclusion, the species was microscopically detected as *Pinus massoniana*, suggesting its applicability to wooden ship making in the Yangtze Basin region as a crucial wood species. Following underground degradation for approximately 700 years, a 41.07–33.85% drop in the cellulose crystallinity was noted for the *Pinus massoniana*, in contrast to the new wood. According to the FTIR pattern differences, there were chemical alterations in the natural aging process, which might have been due to hemicellulose deacetylation. The mechanical properties were also reduced by approximately 20.9–55.6%. As revealed by the morphological trait comparison between the ancient and new wood specimens, most pits were broken during natural aging due to cellulose and hemicellulose deacetylation.

## Figures and Tables

**Table 1:** Nanoindentation test results for ancient wood and new wood samples.

Wood Samples	Elastic Modulus (GPa)	Hardness (GPa)
Ancient wood	5.99 (0.39)	0.20 (0.02)
New wood	7.57 (0.26)	0.45 (0.02) ^1^

^1^ The average values of 25 measurements and their standard deviations are in brackets.

## Data Availability

Not applicable.
